# Mutually Exclusive Interventions in the Cost-Effectiveness Bookshelf

**DOI:** 10.1177/0272989X20912261

**Published:** 2020-03-14

**Authors:** Jonathan Siverskog, Martin Henriksson

**Affiliations:** Centre for Medical Technology Assessment, Linköping University, Linköping, Sweden; Centre for Medical Technology Assessment, Linköping University, Linköping, Sweden

**Keywords:** cost-effectiveness analysis, cost-effectiveness threshold, health economics, opportunity cost, resource allocation

The cost-effectiveness bookshelf^1,2^ is a graphical model that aids the interpretation and discussion of opportunity cost and cost-effectiveness thresholds in health care decision making. It is also an excellent tool for teaching the principles of cost-effectiveness analysis (CEA) since it provides an intuitive illustration of how incremental cost-effectiveness ratios (ICERs) can be used to maximize an outcome subject to a budget constraint. The way in which ICERs are used depends on whether they inform a choice between *independent* interventions (all of the interventions can be adopted with a sufficient budget; e.g., choosing between a cancer drug and a lipid-lowering drug) or *mutually exclusive* interventions (only one of the interventions can be adopted; e.g., choosing between different designs of a screening program).^3^ However, previous accounts of the model have not paid much attention to this difference, which is an important aspect of CEA that often causes confusion.^4^

In this article, we show that the extension of the cost-effectiveness bookshelf to include mutually exclusive interventions makes it necessary to distinguish between average and incremental cost-effectiveness in the model. We demonstrate that the model must be based on incremental cost-effectiveness for the books to be meaningfully ordered on the shelf (i.e., reflect the information relevant to the optimization problem they are supposed to illustrate) and propose an interpretation of the model that allows for such an illustration.

We contribute to previous accounts of the cost-effectiveness bookshelf in 2 important respects. First, since it is important to grasp the difference between independent and mutually exclusive interventions to understand the role of ICERs in constrained maximization, incorporating the concept in the cost-effectiveness bookshelf ought to be helpful for teaching CEA. Second, although the bookshelf is a great teaching tool, some might find it too unrealistic a description of a health care system to be of any real value outside of the classroom. Our account of the bookshelf helps clarify that it may actually be a reasonable way to characterize a health care system. This is because *intervention* can be given a very flexible interpretation, the most important implication of which is that when an intervention is displaced, it need not imply that a health care service is discontinued, merely that it is scaled down in some fashion. In what follows, we begin by briefly reiterating the bookshelf metaphor of a health care system.

## The Bookshelf

Assume that health is measured in quality-adjusted life years (QALYs) and that expenditure is measured in dollars ($). Further assume that health care consists of separate interventions, each represented by a book. The height of a book indicates the number of QALYs the intervention produces per dollar, and the width of a book represents the intervention’s budget impact. Consequently, the area of a book’s spine equals the number of QALYs the intervention produces. All books are kept on a bookshelf, but the health care system faces a budget restriction in the form of a bookend. The books arranged to the left of the bookend are the interventions currently included in the system. The books to the right constitute potential investments, but moving one to the left end of the shelf would force another book out unless the bookend is moved to the right (the budget is increased) to make room for it.

**Figure 1:**
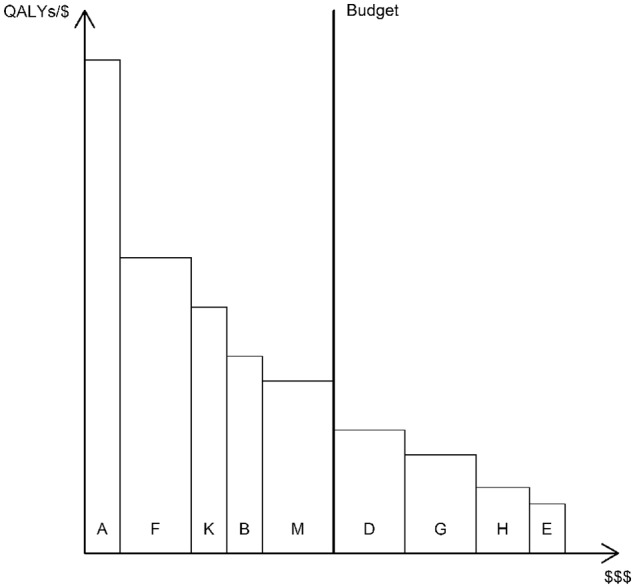
Bookshelf illustration of a health care system with a set of 9 independent interventions from which to choose. QALYs, quality-adjusted life years.

The bookshelf in Figure 1 depicts an imaginary health care system with a constrained budget and a set of 9 independent interventions from which to choose. Health is maximized if we arrange the books according to height in descending order. The first book to the left of the bookend (M) is referred to as the marginal intervention, since it determines the marginal cost of health and is the one that would be displaced by new investments, assuming displacement is efficient. When an intervention is considered for investment, it must be more cost-effective (taller) than M to generate a net health gain. Thus, the bookshelf illustrates that decision making based on a cost-effectiveness threshold is consistent with health maximization and that the health-maximizing threshold is determined by the cost-effectiveness of the marginal intervention.

## Mutually Exclusive Interventions in the Bookshelf

We borrow an existing example of the difference between evaluating independent and mutually exclusive interventions,^4^ the data from which are reported in Table 1, to demonstrate the implications of basing the bookshelf on either average or incremental cost-effectiveness.

**Table 1:** Example^4^ of 11 Interventions for 3 Different Patient Groups with 1000 Patients in Each Group^a^

By Patient Group and Effectiveness					By ICER				
Intervention	Group	C	E	CER	Intervention	Group	ΔC	ΔE	ICER
A	1	100	10	10	A	1	100	10	10
B	1	200	14	14	F	2	200	12	17
C	1	300	16	19	K	3	100	5	20
D	1	400	19	21	B	1	100	4	25
E	1	500	20	25	M	3	200	7	29
F	2	200	12	17	D	1	200	5	40
G	2	400	16	25	G	2	200	4	50
H	2	550	18	31	H	2	150	2	75
K	3	100	5	20	E	1	100	1	100
L	3	200	8	25	C	1			^b^s
M	3	300	12	25	L	3			^b^

a C is the dollar cost per patient. E is the quality-adjusted life years (QALYs) gained per patient. (I)CER is the (incremental) cost-effectiveness ratio of an intervention. All interventions within a patient group are mutually exclusive.

b Extendedly dominated.

Imagine a health care system with a $700,000 budget and a set of 11 interventions from which to choose. Health care may be provided for 3 different patient groups, each consisting of 1000 patients, but all interventions within a group are mutually exclusive. We shall assume that these interventions are divisible, since it makes the optimization problem more straightforward^3,5^ and will be useful to illustrate a particular point (divisibility can be interpreted in terms of only providing an intervention for some of the patients in a group). Health maximization would first lead to the implementation of interventions A, F, and K. Then, intervention A would be replaced by intervention B and intervention K by intervention M, at which point the budget would be exhausted.

**Figure 2:**
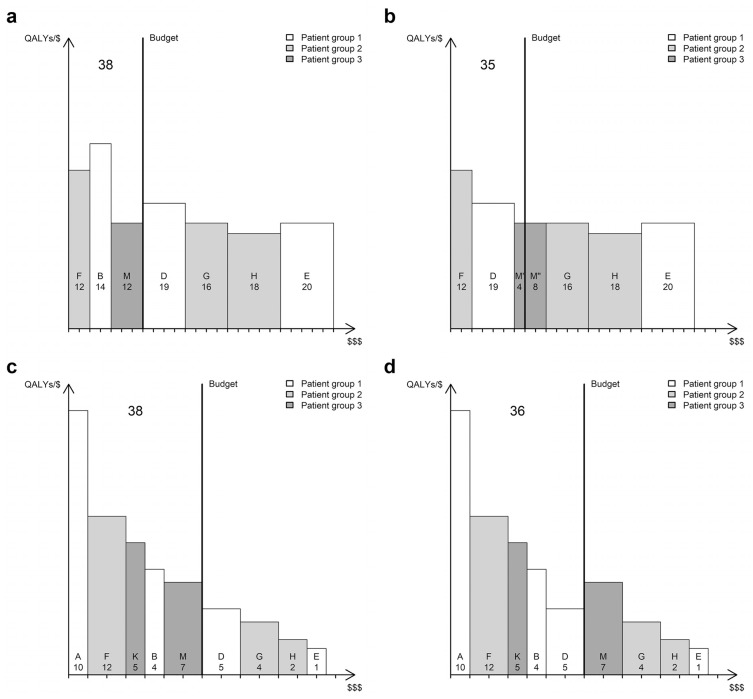
Bookshelf illustrations of a health care system where interventions within a patient group are mutually exclusive. The height and width of a book represents (a, b) average cost-effectiveness and total cost or (c, d) incremental cost-effectiveness and incremental cost. Bookshelves to the left (a and c) depict the reference scenario where the system remains unchanged; bookshelves to the right (b and d) depict the scenario where intervention D is included in the system. A tick on the horizontal axis represents $100,000 in total spending. Numbers represent thousands of quality-adjusted life years (QALYs) produced by the individual interventions (small) and the health care system in total (big).

For some reason, intervention D is now considered for adoption. First, let the height and width of each book represent the average cost-effectiveness and total cost of each intervention, respectively (Figure 2a). If we were to include intervention D, it would replace intervention B (which is removed from the shelf) and displace two-thirds of intervention M, leading to a net loss of 3000 QALYs (Figure 2b). From this illustration, it is not apparent that the books are organized in the order they ought to be picked. Neither is it obvious, by comparing the heights of books D and M, that there would be a net health loss, which illustrates a well-known critique against the use of average cost-effectiveness ratios in decision making.^3^ Furthermore, it arguably misrepresents the displacement that would take place, which is seen when we instead let the height and width of each book represent the incremental cost-effectiveness and incremental cost of each intervention, respectively (Figure 2c). This illustration requires us to recognize that some books belong together as different volumes of the same collection. For instance, intervention B is represented by books A and B, while intervention D is represented by books A, B, and D. In Figure 2d, we see that the inclusion of book D displaces book M. In other words, intervention D is funded by withdrawing intervention M and reverting to intervention K for patient group 3.

It would of course be possible to illustrate the above scenario correctly using average cost-effectiveness (or a mix of average and incremental cost-effectiveness), but picking books by height only mirrors the decision rules for health maximization when the height of all books represents incremental cost-effectiveness. However, note that the bookshelf in Figure 2c is the same as the one in Figure 1. The only modification to the model necessary to accommodate the presence of mutually exclusive interventions is that individual books be given a different interpretation. According to this, an intervention is usually not represented by a single book. For instance, the white books in Figure 2c could represent a screening program where book A is a single screening occasion, book B adds a rescreening after 5 years, and book D adds a further rescreening after 10 years. Therefore, funding a new screening program adds 1 or more books to the shelf, depending on which design we opt for, rather than a single book with adjustable height and width. This interpretation is relevant, not just for screening designs but for most interventions, since there is almost always a choice between mutually exclusive alternatives (e.g., there are several options on how to treat the same condition and they can be reimbursed for several subgroups of patients). Similarly, the inclusion or displacement of a single book could mean that an already funded health care service is expanded or reduced in some fashion (e.g., a change in the frequency of a screening program). Such an expansion or reduction means that we replace one intervention with another but does not necessarily correspond to something that we would usually think of as such (e.g., a change in the number of nurses employed by a care unit). Furthermore, it is important to note that replacing an intervention is not the same as replacing a book; since a book describes the increment in effectiveness and cost of one intervention compared to another, it will never replace the book(s) of the comparator (the exception is when a new intervention [extendedly] dominates existing ones, which would mean that the shape of the affected books would have to be altered).

## Implications and Conclusion

In addition to showing how 2 excellent teaching resources in CEA^1,4^ fit together, our suggested interpretation may help bring the bookshelf model more closely in line with the world of health care decision making with which one might be familiar (cf. Birch and Gafni^6^). It shows that it is not necessary to be able to divide the health care system into individual treatments, technologies, or programs of care for the model to serve as a useful analytic tool, since an intervention can essentially be thought of as an arbitrarily small change to the system.

Our account of the cost-effectiveness bookshelf also clarifies how it may be appropriate to interpret results from the model. One way of expressing its main result is to say that the health-maximizing cost-effectiveness threshold is determined by the ICER of the least cost-effective currently funded health care service. However, the bookshelf (or the theory it illustrates^5^) does not imply that there actually is a single health care service that could be used to determine this threshold. If we attempt to translate from the model to reality, it seems far more plausible to imagine many thin books at and close to the margin, which would all be displaced by a new investment, symbolizing the slight reduction of many different health care services to finance the new one. That it has proved difficult to identify displaced interventions^7–9^ would seem to support such an interpretation. Recent empirical work attempting to estimate the marginal effect of health care spending^10–13^ can be seen as a less literal way of trying to identify the marginal intervention.^14^

## References

[1] Culyer AJ. Cost-effectiveness thresholds in health care: a bookshelf guide to their meaning and use. Health Econ Policy Law. 2016;11(4):415–32. 10.1017/S1744133116000049 26906561

[2] Paulden M O’Mahony J McCabe C. Determinants of change in the cost-effectiveness threshold. Med Decis Making. 2017;37(2):264–76. 10.1177/0272989X16662242 27553208

[3] Johannesson M Weinstein MC. On the decision rules of cost-effectiveness analysis. J Health Econ. 1993;12(4):459–67. 10.1016/0167-6296(93)90005-y 10131756

[4] Karlsson G Johannesson M. The decision rules of cost-effectiveness analysis. Pharmacoeconomics. 1996;9(2):113–20. 10.2165/00019053-199609020-00003 10160090

[5] Weinstein M Zeckhauser R. Critical ratios and efficient allocation. J Public Econ. 1973;2(2):147–57.

[6] Birch S Gafni A. Changing the problem to fit the solution: Johannesson and Weinstein’s (mis) application of economics to real world problems. J Health Econ. 1993;12(4):469–76. 10.1016/0167-6296(93)90006-z 10131757

[7] Karlsberg Schaffer S Sussex J Devlin N Walker A Local health care expenditure plans and their opportunity costs. Health Policy. 2015;119(9):1237–44. 10.1016/j.healthpol.2015.07.007 26251323

[8] Karlsberg Schaffer S Sussex J Hughes D Devlin N Opportunity costs and local health service spending decisions: a qualitative study from Wales. BMC Health Serv Res. 2016;16(1):103. 27012523 10.1186/s12913-016-1354-1 PMC4807555

[9] Appleby J Devlin N Parkin D Buxton M Chalkidou K. Searching for cost effectiveness thresholds in the NHS. Health Policy. 2009;91(3):239–45. 10.1016/j.healthpol.2008.12.010 19168255

[10] Vallejo-Torres L Garcia-Lorenzo B Serrano-Aguilar P. Estimating a cost-effectiveness threshold for the Spanish NHS. Health Econ. 2018;27(4):746–61. 10.1002/hec.3633 29282798

[11] Siverskog J Henriksson M. Estimating the marginal cost of a life year in Sweden’s public healthcare sector. Eur J Health Econ. 2019;20(5):751–62. 10.1007/s10198-019-01039-0 PMC6602994 30796552

[12] Edney LC Afzali HHA Cheng TC Karnon J. Estimating the reference incremental cost-effectiveness ratio for the Australian health system. Pharmacoeconomics. 2018;36(2):239–52. 10.1007/s40273-017-0585-2 29273843

[13] Claxton K Martin S Soares M, et al Methods for the estimation of the National Institute for Health and Care Excellence cost-effectiveness threshold. Health Technol Assess. 2015;19(14):1–503. 10.3310/hta19140 PMC4781395 25692211

[14] Thokala P Ochalek J Leech AA Tong T. Cost-effectiveness thresholds: the past, the present and the future. Pharmacoeconomics. 2018;36(5):509–22. 10.1007/s40273-017-0606-1 29427072

